# Functional annotation of the 2q35 breast cancer risk locus implicates a structural variant in influencing activity of a long-range enhancer element

**DOI:** 10.1016/j.ajhg.2021.05.013

**Published:** 2021-06-18

**Authors:** Joseph S. Baxter, Nichola Johnson, Katarzyna Tomczyk, Andrea Gillespie, Sarah Maguire, Rachel Brough, Laura Fachal, Kyriaki Michailidou, Manjeet K. Bolla, Qin Wang, Joe Dennis, Thomas U. Ahearn, Irene L. Andrulis, Hoda Anton-Culver, Natalia N. Antonenkova, Volker Arndt, Kristan J. Aronson, Annelie Augustinsson, Heiko Becher, Matthias W. Beckmann, Sabine Behrens, Javier Benitez, Marina Bermisheva, Natalia V. Bogdanova, Stig E. Bojesen, Hermann Brenner, Sara Y. Brucker, Qiuyin Cai, Daniele Campa, Federico Canzian, Jose E. Castelao, Tsun L. Chan, Jenny Chang-Claude, Stephen J. Chanock, Georgia Chenevix-Trench, Ji-Yeob Choi, Christine L. Clarke, NBCS Collaborators, Sarah Colonna, Don M. Conroy, Fergus J. Couch, Angela Cox, Simon S. Cross, Kamila Czene, Mary B. Daly, Peter Devilee, Thilo Dörk, Laure Dossus, Miriam Dwek, Diana M. Eccles, Arif B. Ekici, A. Heather Eliassen, Christoph Engel, Peter A. Fasching, Jonine Figueroa, Henrik Flyger, Manuela Gago-Dominguez, Chi Gao, Montserrat García-Closas, José A. García-Sáenz, Maya Ghoussaini, Graham G. Giles, Mark S. Goldberg, Anna González-Neira, Pascal Guénel, Melanie Gündert, Lothar Haeberle, Eric Hahnen, Christopher A. Haiman, Per Hall, Ute Hamann, Mikael Hartman, Sigrid Hatse, Jan Hauke, Antoinette Hollestelle, Reiner Hoppe, John L. Hopper, Ming-Feng Hou, kConFab Investigators, ABCTB Investigators, Hidemi Ito, Motoki Iwasaki, Agnes Jager, Anna Jakubowska, Wolfgang Janni, Esther M. John, Vijai Joseph, Audrey Jung, Rudolf Kaaks, Daehee Kang, Renske Keeman, Elza Khusnutdinova, Sung-Won Kim, Veli-Matti Kosma, Peter Kraft, Vessela N. Kristensen, Katerina Kubelka-Sabit, Allison W. Kurian, Ava Kwong, James V. Lacey, Diether Lambrechts, Nicole L. Larson, Susanna C. Larsson, Loic Le Marchand, Flavio Lejbkowicz, Jingmei Li, Jirong Long, Artitaya Lophatananon, Jan Lubiński, Arto Mannermaa, Mehdi Manoochehri, Siranoush Manoukian, Sara Margolin, Keitaro Matsuo, Dimitrios Mavroudis, Rebecca Mayes, Usha Menon, Roger L. Milne, Nur Aishah Mohd Taib, Kenneth Muir, Taru A. Muranen, Rachel A. Murphy, Heli Nevanlinna, Katie M. O’Brien, Kenneth Offit, Janet E. Olson, Håkan Olsson, Sue K. Park, Tjoung-Won Park-Simon, Alpa V. Patel, Paolo Peterlongo, Julian Peto, Dijana Plaseska-Karanfilska, Nadege Presneau, Katri Pylkäs, Brigitte Rack, Gad Rennert, Atocha Romero, Matthias Ruebner, Thomas Rüdiger, Emmanouil Saloustros, Dale P. Sandler, Elinor J. Sawyer, Marjanka K. Schmidt, Rita K. Schmutzler, Andreas Schneeweiss, Minouk J. Schoemaker, Mitul Shah, Chen-Yang Shen, Xiao-Ou Shu, Jacques Simard, Melissa C. Southey, Jennifer Stone, Harald Surowy, Anthony J. Swerdlow, Rulla M. Tamimi, William J. Tapper, Jack A. Taylor, Soo Hwang Teo, Lauren R. Teras, Mary Beth Terry, Amanda E. Toland, Ian Tomlinson, Thérèse Truong, Chiu-Chen Tseng, Michael Untch, Celine M. Vachon, Ans M.W. van den Ouweland, Sophia S. Wang, Clarice R. Weinberg, Camilla Wendt, Stacey J. Winham, Robert Winqvist, Alicja Wolk, Anna H. Wu, Taiki Yamaji, Wei Zheng, Argyrios Ziogas, Paul D.P. Pharoah, Alison M. Dunning, Douglas F. Easton, Stephen J. Pettitt, Christopher J. Lord, Syed Haider, Nick Orr, Olivia Fletcher

**Affiliations:** 1 The Breast Cancer Now Toby Robins Research Centre, The Institute of Cancer Research, London SW7 3RP, UK; 2 Centre for Cancer Research and Cell Biology, Queen’s University Belfast, Belfast, Ireland BT7 1NN, UK; 3 The CRUK Gene Function Laboratory, The Institute of Cancer Research, London SW3 6JB, UK; 4 Centre for Cancer Genetic Epidemiology, Department of Oncology, University of Cambridge, Cambridge CB1 8RN, UK; 5 Biostatistics Unit, The Cyprus Institute of Neurology & Genetics, Nicosia 2371, Cyprus; 6 Cyprus School of Molecular Medicine, The Cyprus Institute of Neurology & Genetics, Nicosia 2371, Cyprus; 7 Centre for Cancer Genetic Epidemiology, Department of Public Health and Primary Care, University of Cambridge, Cambridge CB1 8RN, UK; 8 Division of Cancer Epidemiology and Genetics, National Cancer Institute, National Institutes of Health, Department of Health and Human Services, Bethesda, MD 20850, USA; 9 Fred A. Litwin Center for Cancer Genetics, Lunenfeld-Tanenbaum Research Institute of Mount Sinai Hospital, Toronto, ON M5G 1X5, Canada; 10 Department of Molecular Genetics, University of Toronto, Toronto, ON M5S 1A8, Canada; 11 Department of Medicine, Genetic Epidemiology Research Institute, University of California Irvine, Irvine, CA 92617, USA; 12 N.N. Alexandrov Research Institute of Oncology and Medical Radiology, Minsk 223040, Belarus; 13 Division of Clinical Epidemiology and Aging Research, German Cancer Research Center (DKFZ), Heidelberg 69120, Germany; 14 Department of Public Health Sciences, and Cancer Research Institute, Queen’s University, Kingston, ON K7L 3N6, Canada; 15 Department of Cancer Epidemiology, Clinical Sciences, Lund University, Lund 222 42, Sweden; 16 Institute of Medical Biometry and Epidemiology, University Medical Center Hamburg-Eppendorf, Hamburg 20246, Germany; 17 Department of Gynecology and Obstetrics, Comprehensive Cancer Center Erlangen-EMN, University Hospital Erlangen, Friedrich-Alexander-University Erlangen-Nuremberg (FAU), Erlangen 91054, Germany; 18 Division of Cancer Epidemiology, German Cancer Research Center (DKFZ), Heidelberg 69120, Germany; 19 Biomedical Network on Rare Diseases (CIBERER), Madrid 28029, Spain; 20 Human Cancer Genetics Programme, Spanish National Cancer Research Centre (CNIO), Madrid 28029, Spain; 21 Institute of Biochemistry and Genetics, Ufa Federal Research Centre of the Russian Academy of Sciences, Ufa 450054, Russia; 22 Department of Radiation Oncology, Hannover Medical School, Hannover 30625, Germany; 23 Gynaecology Research Unit, Hannover Medical School, Hannover 30625, Germany; 24 Copenhagen General Population Study, Herlev and Gentofte Hospital, Copenhagen University Hospital, Herlev 2730, Denmark; 25 Department of Clinical Biochemistry, Herlev and Gentofte Hospital, Copenhagen University Hospital, Herlev 2730, Denmark; 26 Faculty of Health and Medical Sciences, University of Copenhagen, Copenhagen 2200, Denmark; 27 Division of Preventive Oncology, German Cancer Research Center (DKFZ) and National Center for Tumor Diseases (NCT), Heidelberg 69120, Germany; 28 German Cancer Consortium (DKTK), German Cancer Research Center (DKFZ), Heidelberg 69120, Germany; 29 Department of Gynecology and Obstetrics, University of Tübingen, Tübingen 72076, Germany; 30 Division of Epidemiology, Department of Medicine, Vanderbilt Epidemiology Center, Vanderbilt-Ingram Cancer Center, Vanderbilt University School of Medicine, Nashville, TN 37232, USA; 31 Department of Biology, University of Pisa, Pisa 56126, Italy; 32 Genomic Epidemiology Group, German Cancer Research Center (DKFZ), Heidelberg 69120, Germany; 33 Oncology and Genetics Unit, Instituto de Investigación Sanitaria Galicia Sur (IISGS), Xerencia de Xestion Integrada de Vigo-SERGAS, Vigo 36312, Spain; 34 Hong Kong Hereditary Breast Cancer Family Registry, Hong Kong; 35 Department of Molecular Pathology, Hong Kong Sanatorium and Hospital, Hong Kong; 36 Cancer Epidemiology Group, University Cancer Center Hamburg (UCCH), University Medical Center Hamburg-Eppendorf, Hamburg 20246, Germany; 37 Department of Genetics and Computational Biology, QIMR Berghofer Medical Research Institute, Brisbane, QLD 4006, Australia; 38 Department of Biomedical Sciences, Seoul National University Graduate School, Seoul 03080, Korea; 39 Cancer Research Institute, Seoul National University, Seoul 03080, Korea; 40 Institute of Health Policy and Management, Seoul National University Medical Research Center, Seoul 03080, Korea; 41 Westmead Institute for Medical Research, University of Sydney, Sydney, NSW 2145, Australia; 42 Department of Cancer Genetics, Institute for Cancer Research, Oslo University Hospital-Radiumhospitalet, Oslo 0379, Norway; 43 Institute of Clinical Medicine, Faculty of Medicine, University of Oslo, Oslo 0450, Norway; 44 Department of Research, Vestre Viken Hospital, Drammen 3019, Norway; 45 Section for Breast and Endocrine Surgery, Department of Cancer, Division of Surgery, Cancer and Transplantation Medicine, Oslo University Hospital-Ullevål, Oslo 0450, Norway; 46 Department of Radiology and Nuclear Medicine, Oslo University Hospital, Oslo 0379, Norway; 47 Department of Pathology, Akershus University Hospital, Lørenskog 1478, Norway; 48 Department of Tumor Biology, Institute for Cancer Research, Oslo University Hospital, Oslo 0379, Norway; 49 Department of Oncology, Division of Surgery, Cancer and Transplantation Medicine, Oslo University Hospital-Radiumhospitalet, Oslo 0379, Norway; 50 National Advisory Unit on Late Effects after Cancer Treatment, Oslo University Hospital-Radiumhospitalet, Oslo 0379, Norway; 51 Department of Oncology, Akershus University Hospital, Lørenskog 1478, Norway; 52 Breast Cancer Research Consortium, Oslo University Hospital, Oslo 0379, Norway; 53 Department of Medicine, Huntsman Cancer Institute, Salt Lake City, UT 84112, USA; 54 Department of Laboratory Medicine and Pathology, Mayo Clinic, Rochester, MN 55905, USA; 55 Sheffield Institute for Nucleic Acids (SInFoNiA), Department of Oncology and Metabolism, University of Sheffield, Sheffield S10 2TN, UK; 56 Academic Unit of Pathology, Department of Neuroscience, University of Sheffield, Sheffield S10 2TN, UK; 57 Department of Medical Epidemiology and Biostatistics, Karolinska Institutet, Stockholm 171 65, Sweden; 58 Department of Clinical Genetics, Fox Chase Cancer Center, Philadelphia, PA 19111, USA; 59 Department of Pathology, Leiden University Medical Center, Leiden 2333 ZA, the Netherlands; 60 Department of Human Genetics, Leiden University Medical Center, Leiden 2333 ZA, the Netherlands; 61 Nutrition and Metabolism Section, International Agency for Research on Cancer (IARC-WHO), Lyon 69372, France; 62 School of Life Sciences, University of Westminster, London W1B 2HW, UK; 63 Faculty of Medicine, University of Southampton, Southampton SO17 1BJ, UK; 64 Institute of Human Genetics, University Hospital Erlangen, Friedrich-Alexander University Erlangen-Nuremberg, Comprehensive Cancer Center Erlangen-EMN, Erlangen 91054, Germany; 65 Channing Division of Network Medicine, Department of Medicine, Brigham and Women’s Hospital and Harvard Medical School, Boston, MA 02115, USA; 66 Department of Epidemiology, Harvard T.H. Chan School of Public Health, Boston, MA 02115, USA; 67 Institute for Medical Informatics, Statistics and Epidemiology, University of Leipzig, Leipzig 04107, Germany; 68 LIFE - Leipzig Research Centre for Civilization Diseases, University of Leipzig, Leipzig 04103, Germany; 69 David Geffen School of Medicine, Department of Medicine Division of Hematology and Oncology, University of California at Los Angeles, Los Angeles, CA 90095, USA; 70 Usher Institute of Population Health Sciences and Informatics, The University of Edinburgh, Edinburgh EH16 4UX, UK; 71 Cancer Research UK Edinburgh Centre, The University of Edinburgh, Edinburgh EH4 2XR, UK; 72 Department of Breast Surgery, Herlev and Gentofte Hospital, Copenhagen University Hospital, Herlev 2730, Denmark; 73 Fundación Pública Galega de Medicina Xenómica, Instituto de Investigación Sanitaria de Santiago de Compostela (IDIS), Complejo Hospitalario Universitario de Santiago, SERGAS, Santiago de Compostela 15706, Spain; 74 Moores Cancer Center, University of California San Diego, La Jolla, CA 92037, USA; 75 Program in Genetic Epidemiology and Statistical Genetics, Harvard T.H. Chan School of Public Health, Boston, MA 02115, USA; 76 Medical Oncology Department, Hospital Clínico San Carlos, Instituto de Investigación Sanitaria San Carlos (IdISSC), Centro Investigación Biomédica en Red de Cáncer (CIBERONC), Madrid 28040, Spain; 77 Open Targets, Core Genetics Team, Wellcome Sanger Institute, Hinxton, Cambridge CB10 1SA, UK; 78 Cancer Epidemiology Division, Cancer Council Victoria, Melbourne, VIC 3004, Australia; 79 Centre for Epidemiology and Biostatistics, Melbourne School of Population and Global Health, The University of Melbourne, Melbourne, VIC 3010, Australia; 80 Precision Medicine, School of Clinical Sciences at Monash Health, Monash University, Clayton, VIC 3168, Australia; 81 Department of Medicine, McGill University, Montréal, QC H4A 3J1, Canada; 82 Division of Clinical Epidemiology, Royal Victoria Hospital, McGill University, Montréal, QC H4A 3J1, Canada; 83 Center for Research in Epidemiology and Population Health (CESP), Team Exposome and Heredity, INSERM, University Paris-Saclay, Villejuif 94805, France; 84 Molecular Epidemiology Group, C080, German Cancer Research Center (DKFZ), Heidelberg 69120, Germany; 85 Molecular Biology of Breast Cancer, University Womens Clinic Heidelberg, University of Heidelberg, Heidelberg 69120, Germany; 86 Institute of Diabetes Research, Helmholtz Zentrum München, German Research Center for Environmental Health, Neuherberg 85764, Germany; 87 Center for Familial Breast and Ovarian Cancer, Faculty of Medicine and University Hospital Cologne, University of Cologne, Cologne 50937, Germany; 88 Center for Integrated Oncology (CIO), Faculty of Medicine and University Hospital Cologne, University of Cologne, Cologne 50937, Germany; 89 Department of Preventive Medicine, Keck School of Medicine, University of Southern California, Los Angeles, CA 90033, USA; 90 Department of Oncology, Södersjukhuset, Stockholm 118 83, Sweden; 91 Molecular Genetics of Breast Cancer, German Cancer Research Center (DKFZ), Heidelberg 69120, Germany; 92 Saw Swee Hock School of Public Health, National University of Singapore, Singapore 119077, Singapore; 93 Department of Surgery, National University Hospital, Singapore 119228, Singapore; 94 Yong Loo Lin School of Medicine, National University of Singapore, Singapore 119077, Singapore; 95 Laboratory of Experimental Oncology (LEO), Department of Oncology, KU Leuven, Leuven Cancer Institute, Leuven 3000, Belgium; 96 Center for Molecular Medicine Cologne (CMMC), Faculty of Medicine and University Hospital Cologne, University of Cologne, Cologne 50931, Germany; 97 Department of Medical Oncology, Erasmus MC Cancer Institute, Rotterdam 3015 GD, the Netherlands; 98 Dr. Margarete Fischer-Bosch-Institute of Clinical Pharmacology, Stuttgart 70376, Germany; 99 University of Tübingen, Tübingen 72074, Germany; 100 Department of Surgery, Kaohsiung Municipal Hsiao-Kang Hospital, Kaohsiung 812, Taiwan; 101 Research Department, Peter MacCallum Cancer Center, Melbourne, VIC 3000, Australia; 102 Sir Peter MacCallum Department of Oncology, The University of Melbourne, Melbourne, VIC 3000, Australia; 103 Australian Breast Cancer Tissue Bank, Westmead Institute for Medical Research, University of Sydney, Sydney, NSW 2145, Australia; 104 Division of Cancer Epidemiology and Prevention, Aichi Cancer Center Research Institute, Nagoya 464-8681, Japan; 105 Division of Cancer Epidemiology, Nagoya University Graduate School of Medicine, Nagoya 466-8550, Japan; 106 Division of Epidemiology, Center for Public Health Sciences, National Cancer Center, Tokyo 104-0045, Japan; 107 Department of Genetics and Pathology, Pomeranian Medical University, Szczecin 71-252, Poland; 108 Independent Laboratory of Molecular Biology and Genetic Diagnostics, Pomeranian Medical University, Szczecin 71-252, Poland; 109 Department of Gynaecology and Obstetrics, University Hospital Ulm, Ulm 89075, Germany; 110 Department of Epidemiology & Population Health, Stanford University School of Medicine, Stanford, CA 94305, USA; 111 Department of Medicine, Division of Oncology, Stanford Cancer Institute, Stanford University School of Medicine, Stanford, CA 94304, USA; 112 Clinical Genetics Research Lab, Department of Cancer Biology and Genetics, Memorial Sloan Kettering Cancer Center, New York, NY 10065, USA; 113 Department of Preventive Medicine, Seoul National University College of Medicine, Seoul 03080, Korea; 114 Division of Molecular Pathology, the Netherlands Cancer Institute - Antoni van Leeuwenhoek Hospital, Amsterdam 1066 CX, the Netherlands; 115 Department of Genetics and Fundamental Medicine, Bashkir State University, Ufa 450000, Russia; 116 Department of Surgery, Daerim Saint Mary’s Hospital, Seoul 07442, Korea; 117 Translational Cancer Research Area, University of Eastern Finland, Kuopio 70210, Finland; 118 Institute of Clinical Medicine, Pathology and Forensic Medicine, University of Eastern Finland, Kuopio 70210, Finland; 119 Biobank of Eastern Finland, Kuopio University Hospital, Kuopio, Finland; 120 Department of Medical Genetics, Oslo University Hospital and University of Oslo, Oslo 0379, Norway; 121 Department of Histopathology and Cytology, Clinical Hospital Acibadem Sistina, Skopje 1000, Republic of North Macedonia; 122 Department of Surgery, The University of Hong Kong, Hong Kong; 123 Department of Surgery and Cancer Genetics Center, Hong Kong Sanatorium and Hospital, Hong Kong; 124 Department of Computational and Quantitative Medicine, City of Hope, Duarte, CA 91010, USA; 125 City of Hope Comprehensive Cancer Center, City of Hope, Duarte, CA 91010, USA; 126 VIB Center for Cancer Biology, Leuven 3001, Belgium; 127 Laboratory for Translational Genetics, Department of Human Genetics, University of Leuven, Leuven 3000, Belgium; 128 Department of Health Sciences Research, Mayo Clinic, Rochester, MN 55905, USA; 129 Institute of Environmental Medicine, Karolinska Institutet, Stockholm 171 77, Sweden; 130 Department of Surgical Sciences, Uppsala University, Uppsala 751 05, Sweden; 131 Epidemiology Program, University of Hawaii Cancer Center, Honolulu, HI 96813, USA; 132 Clalit National Cancer Control Center, Carmel Medical Center and Technion Faculty of Medicine, Haifa 35254, Israel; 133 Human Genetics Division, Genome Institute of Singapore, Singapore 138672, Singapore; 134 Division of Population Health, Health Services Research and Primary Care, School of Health Sciences, Faculty of Biology, Medicine and Health, The University of Manchester, Manchester M13 9PL, UK; 135 Unit of Medical Genetics, Department of Medical Oncology and Hematology, Fondazione IRCCS Istituto Nazionale dei Tumori di Milano, Milan 20133, Italy; 136 Department of Clinical Science and Education, Södersjukhuset, Karolinska Institutet, Stockholm 118 83, Sweden; 137 Department of Medical Oncology, University Hospital of Heraklion, Heraklion 711 10, Greece; 138 Institute of Clinical Trials & Methodology, University College London, London WC1V 6LJ, UK; 139 Breast Cancer Research Unit, University Malaya Cancer Research Institute, Faculty of Medicine, University of Malaya, Kuala Lumpur 50603, Malaysia; 140 Department of Obstetrics and Gynecology, Helsinki University Hospital, University of Helsinki, Helsinki 00290, Finland; 141 School of Population and Public Health, University of British Columbia, Vancouver, BC V6T 1Z4, Canada; 142 Cancer Control Research, BC Cancer, Vancouver, BC V5Z 1L3, Canada; 143 Epidemiology Branch, National Institute of Environmental Health Sciences, NIH, Research Triangle Park, NC 27709, USA; 144 Clinical Genetics Service, Department of Medicine, Memorial Sloan Kettering Cancer Center, New York, NY 10065, USA; 145 Convergence Graduate Program in Innovative Medical Science, Seoul National University College of Medicine, Seoul 03080, Korea; 146 Department of Population Science, American Cancer Society, Atlanta, GA 30303, USA; 147 Genome Diagnostics Program, IFOM - the FIRC Institute of Molecular Oncology, Milan 20139, Italy; 148 Department of Non-Communicable Disease Epidemiology, London School of Hygiene and Tropical Medicine, London WC1E 7HT, UK; 149 Research Centre for Genetic Engineering and Biotechnology ‘Georgi D. Efremov’, MASA, Skopje 1000, Republic of North Macedonia; 150 Laboratory of Cancer Genetics and Tumor Biology, Cancer and Translational Medicine Research Unit, Biocenter Oulu, University of Oulu, Oulu 90570, Finland; 151 Laboratory of Cancer Genetics and Tumor Biology, Northern Finland Laboratory Centre Oulu, Oulu 90570, Finland; 152 Medical Oncology Department, Hospital Universitario Puerta de Hierro, Madrid 28222, Spain; 153 Institute of Pathology, Staedtisches Klinikum Karlsruhe, Karlsruhe 76133, Germany; 154 Department of Oncology, University Hospital of Larissa, Larissa 411 10, Greece; 155 School of Cancer & Pharmaceutical Sciences, Comprehensive Cancer Centre, Guy’s Campus, King’s College London, London, UK; 156 Division of Psychosocial Research and Epidemiology, the Netherlands Cancer Institute - Antoni van Leeuwenhoek hospital, Amsterdam 1066 CX, the Netherlands; 157 National Center for Tumor Diseases, University Hospital and German Cancer Research Center, Heidelberg 69120, Germany; 158 Division of Genetics and Epidemiology, The Institute of Cancer Research, London SM2 5NG, UK; 159 Institute of Biomedical Sciences, Academia Sinica, Taipei 115, Taiwan; 160 School of Public Health, China Medical University, Taichung, Taiwan; 161 Genomics Center, Centre Hospitalier Universitaire de Québec - Université Laval Research Center, Québec City, QC G1V 4G2, Canada; 162 Department of Clinical Pathology, The University of Melbourne, Melbourne, VIC 3010, Australia; 163 Genetic Epidemiology Group, School of Population and Global Health, University of Western Australia, Perth, WA 6000, Australia; 164 Division of Breast Cancer Research, The Institute of Cancer Research, London SW7 3RP, UK; 165 Department of Population Health Sciences, Weill Cornell Medicine, New York, NY 10065, USA; 166 Epigenetic and Stem Cell Biology Laboratory, National Institute of Environmental Health Sciences, NIH, Research Triangle Park, NC 27709, USA; 167 Breast Cancer Research Programme, Cancer Research Malaysia, Subang Jaya, Selangor 47500, Malaysia; 168 Department of Surgery, Faculty of Medicine, University of Malaya, Kuala Lumpur 50603, Malaysia; 169 Department of Epidemiology, Mailman School of Public Health, Columbia University, New York, NY 10032, USA; 170 Department of Cancer Biology and Genetics, The Ohio State University, Columbus, OH 43210, USA; 171 Institute of Cancer and Genomic Sciences, University of Birmingham, Birmingham B15 2TT, UK; 172 Wellcome Trust Centre for Human Genetics and Oxford NIHR Biomedical Research Centre, University of Oxford, Oxford OX3 7BN, UK; 173 Department of Gynecology and Obstetrics, Helios Clinics Berlin-Buch, Berlin 13125, Germany; 174 Department of Health Science Research, Division of Epidemiology, Mayo Clinic, Rochester, MN 55905, USA; 175 Department of Clinical Genetics, Erasmus University Medical Center, Rotterdam 3015 GD, the Netherlands; 176 Biostatistics and Computational Biology Branch, National Institute of Environmental Health Sciences, NIH, Research Triangle Park, NC 27709, USA; 177 Department of Health Sciences Research, Division of Biomedical Statistics and Informatics, Mayo Clinic, Rochester, MN 55905, USA

**Keywords:** breast cancer risk, functional annotation, risk locus

## Abstract

A combination of genetic and functional approaches has identified three independent breast cancer risk loci at 2q35. A recent fine-scale mapping analysis to refine these associations resulted in 1 (signal 1), 5 (signal 2), and 42 (signal 3) credible causal variants at these loci. We used publicly available *in silico* DNase I and ChIP-seq data with *in vitro* reporter gene and CRISPR assays to annotate signals 2 and 3. We identified putative regulatory elements that enhanced cell-type-specific transcription from the *IGFBP5* promoter at both signals (30- to 40-fold increased expression by the putative regulatory element at signal 2, 2- to 3-fold by the putative regulatory element at signal 3). We further identified one of the five credible causal variants at signal 2, a 1.4 kb deletion (esv3594306), as the likely causal variant; the deletion allele of this variant was associated with an average additional increase in *IGFBP5* expression of 1.3-fold (MCF-7) and 2.2-fold (T-47D). We propose a model in which the deletion allele of esv3594306 juxtaposes two transcription factor binding regions (annotated by estrogen receptor alpha ChIP-seq peaks) to generate a single extended regulatory element. This regulatory element increases cell-type-specific expression of the tumor suppressor gene *IGFBP5* and, thereby, reduces risk of estrogen receptor-positive breast cancer (odds ratio = 0.77, 95% CI 0.74–0.81, p = 3.1 × 10^−31^).

## Introduction

Over the last 15 years, genome-wide association studies have transformed our ability to map genetic variation underlying complex traits.^1^ The vast majority of variants identified in genome-wide association studies are non-coding and are thought to influence transcriptional regulation,^2^^,^^3^ a process which can be highly cell type and tissue specific.^4^ Our ability to translate these findings into a greater understanding of the mechanisms that influence an individual woman’s risk will require the identification of causal variants (as opposed to correlative variants), the targets of these functional variants (the genes or non-coding RNAs that mediate the associations observed in genome-wide association studies) and an understanding of the disease causal cell types and processes.^1^ Genome-wide association studies of breast cancer coupled with large-scale replication and fine-mapping studies have led to the identification of approximately 200 breast cancer risk loci;^3^^,^^5^, ^6^, ^7^, ^8^, ^9^ two of these loci, annotated by rs13387042^10^ and rs16857609,^5^ map to a gene desert at chromosome 2q35. Fine-scale mapping, combined with *in silico* annotation, reporter gene assays, and allele-specific qRT-PCR led to the identification of a putative causal variant (rs4442975) at the rs13387042 locus.^11^^,^^12^ rs4442975, which is highly correlated with the tag SNP rs13387042 (r^2^ = 0.92, D′ = 0.96), maps to a consensus binding site for the transcription factor (TF) forkhead box A1 (FOXA1 [MIM: 602294]) with the alternative T-allele promoting binding of FOXA1.^11^^,^^12^ To date, no putative causal variant at the rs16857609 locus has been reported. Chromatin interaction methods implicate *IGFBP5* (MIM: 146734) as the target gene at both loci^11^, ^12^, ^13^ and for the rs13387042 locus, eQTL analyses demonstrated association of the protective T-allele with slightly increased *IGFBP5* levels in normal breast tissue^11^ and estrogen receptor-positive (ER^+^) breast cancers.^12^

Taking a functional approach based on chromosome conformation capture (3C) assays that were anchored at the *IGFBP5* promoter, Wyszynski and colleagues identified a putative regulatory element centered on a structural variant (SV; esv3594306) that maps approximately 400 kb telomeric to *IGFBP5*.^14^ Allele-specific expression analyses and follow-up genotyping identified 14 highly correlated variants (all r^2^ > 0.8 with the top SNP, rs34005590) associated with breast cancer risk, which represent a third risk signal (OR = 0.82, p = 5.6 × 10^−17^).^14^

In this analysis we report fine-scale mapping of the 2q35 region in European and Asian individuals with breast cancer and control subjects from the Breast Cancer Association Consortium. We confirm three independent, high-confidence signals at 2q35 annotated by rs13387042 (signal 1), rs138522813 (signal 2), and rs16857609 (signal 3). We carry out functional annotation of credible variants at signals 2 and 3 and implicate the deletion variant (esv3594306) at signal 2 as causally associated with increased *IGFBP5* expression and reduced breast cancer risk.

## Material and methods

### Fine-scale mapping of the 2q35 breast cancer risk locus

Fine-scale mapping of the 2q35 breast cancer risk locus was carried out as part of a large collaborative project; full details have been published.^3^ Briefly, for the current analysis we accessed data from 94,391 individuals with invasive breast cancer and 83,477 individuals of European ancestry and 12,481 individuals with invasive breast cancer and 12,758 control subjects of Asian ancestry from 87 studies participating in the Breast Cancer Association Consortium. All participating studies were approved by their appropriate ethics review board and all subjects provided informed consent.

Directly genotyped or imputed (info score > 0.8) calls for 10,314 SNPs mapping to a 1.4 Mb region at 2q35 (chr2:217,405,832–218,796,508; GRCh37/hg19) were available for analysis. At this threshold, the proportions of common variants (MAF ≥ 0.05), low-frequency variants (0.01 ≤ MAF < 0.05), and rare variants (0.001 ≤ MAF < 0.01)^3^ that could be analyzed were 89.7%, 68.5%, and 3.6%, respectively, for OncoArray and 64.2%, 40.5%, and 0.8%, respectively, for iCOGS. Analysis of the association between each SNP and risk of breast cancer was performed using unconditional logistic regression assuming a log-additive genetic model, adjusted for study and up to 15 ancestry-informative principal components. p values were calculated using Wald tests. Forward stepwise logistic regression was used to explore whether additional loci in the fine-mapping region were independently associated with breast cancer risk. We carried out stratified analyses to determine whether each of the independent associations differed according to estrogen receptor (ER) status; heterogeneity between stratum-specific estimates was assessed using Cochran’s Q-test. All statistical analyses were carried out using R version 3.6.1.

### *In silico* annotation of credible variants

Credible variants at each of the three independent signals were aligned with DNase I and ChIP-seq data (P300 [EP300 (MIM: 602700)], H3K27Ac, H3K4me1, FOXA1, GATA3 [MIM: 131320], ERα [ESR1 (MIM: 133430)]) generated in T-47D and MCF-7 breast cancer cells^15^, ^16^, ^17^ (Table S1).

### Cloning of reporter assay constructs

All reporter assay plasmids were derived using the pGL4 reporter vector (Promega). Reporter vectors were constructed using a restriction digest-based cloning approach. The *IGFBP5* promoter and putative regulatory element regions (containing WT alleles) were synthesized as gBlocks (Integrated DNA Technologies, full details in Table S2). Double restriction digests of plasmid or gBlock were performed using BglII and XhoI (for *IGFBP5* promoter) or SalI and BamHI (for putative regulatory element regions) according to the manufacturer’s instructions (New England Biolabs [NEB]). Ligations were performed in a 3:1 insert:vector ratio using T4 DNA ligase (NEB), according to manufacturer’s instructions. Correct cloning was validated by Sanger sequencing using a commercially available service (Eurofins Genomics). Alternative (ALT) alleles of each variant were introduced into reporter vectors using QuikChange Lightning Site-directed Mutagenesis kit (Agilent Technologies), according to the manufacturer’s instructions. Accurate mutagenesis was confirmed by Sanger sequencing (Eurofins Genomics). All reporter gene constructs are shown in Figure S1.

### Cell Culture

T-47D cells were grown in RPMI (GIBCO) supplemented with 10% FBS (GIBCO), 10 μg/mL human insulin (Sigma), and 100 U/mL penicillin with 100 μg/mL streptomycin (Sigma). HCT116 cells were grown in RPMI supplemented with 10% FBS, 100 U/mL penicillin, and 100 μg/mL streptomycin. HepG2 cells were grown in EMEM (LGC Standards-ATCC) supplemented with 10% FBS and 100 U/mL penicillin with 100 μg/mL streptomycin. MCF-7 cells (including derivative Cas9-expressing cell lines) and 293T cells were grown in DMEM (GIBCO) supplemented with 10% FBS and 100 U/mL penicillin with 100 μg/mL streptomycin. All cell lines were routinely short tandem repeat (STR)-typed and tested for mycoplasma contamination.

### Reporter assays

Reporter assays were performed in T-47D, MCF-7, 293T, HCT116, and HepG2 cell lines. Antibiotics were removed from standard growth media 24 h before transfection to improve viability. For assays performed under standard conditions, approximately 16,000 cells were seeded per well of a 96-well plate for T-47D, MCF-7, and HepG2, and approximately 8,000 cells were seeded per well of a 96-well plate for 293T and HCT116. Transfection was performed upon reaching 70% confluency (~24 h after cell seeding). For assays performed after 17β-estradiol treatment, cells were first hormone starved for 48 h. Approximately 10,000 cells (T-47D) and 8,000 cells (MCF-7) were seeded, per well of a 96-well plate, in standard growth media and cultured for 24 h. The media was then replaced with phenol red-free media (GIBCO) supplemented with 10% charcoal-stripped FBS (GIBCO), 100 U/mL penicillin with 100 μg/mL streptomycin, 10 nM fulvestrant (I4409, Sigma), and 10 μg/mL human insulin (T-47D only). After 48 h, growth media was replaced with phenol red-free media supplemented with 10% charcoal-stripped FBS, 10 μg/mL human insulin (T-47D only), with the addition of either (1) 10 nM 17β-estradiol (E2758, Sigma) or (2) vehicle (ethanol). Transfection was performed upon reaching 80% confluency (6 h after 17β-estradiol or vehicle treatment).

Transfection was performed using X-treme GENE HP DNA transfection reagent (Roche). Equimolar amounts of the test pGL4-based firefly luciferase vector and pRL-TK renilla luciferase control (Promega) were combined in a 3:1 reagent:DNA ratio in OptiMEM (Fisher Scientific). After a 30 min incubation at room temperature, 10 μL transfection mixture was added per well. Each biological replicate was performed in technical triplicates with non-transfected, mock-transfected, and pEGFP-transfected controls (Takara Bio Inc). Cells were screened for luciferase activity 48 h after transfection using the Dual-Glo Luciferase Assay System (Promega) according to the manufacturer’s instructions.

### Confirmatory genotyping and sequencing of putative regulatory element 2 (PRE2)

Four of the five variants mapping to PRE2 (rs72951831, rs199804270, rs138522813, and esv3594306) are highly correlated based on 1000 Genomes data (1KGP), with the ALT alleles of rs72951831, rs199804270, and rs138522813 all predicted to occur in combination with the ALT (deletion) allele of esv3594306 (esv3594306: rs72951831 r^2^ = 1.0, D′ = 1.0; esv3594306: rs199804270 r^2^ = 0.95, D′ = 1.0; esv3594306: rs138522813 r^2^ = 1.0, D′ = 1.0) . However, rs572022984 (hg19, chr2:217955897) theoretically maps within the esv3594306 deleted region (chr2:217,955,891–217,957,273) casting doubt on whether the (imputed) rs572022984-del allele could occur in combination with the esv3594306 deletion allele. To clarify this, we genotyped all five variants in 300 randomly selected women participating in the Generations Study^18^ using MassARRAY (Agena Bioscience; full details of primers available on request). The number of carriers of the alternative (A>-) allele at rs572022984 (MAF = 0.035) was 0 (expected number = 21; p = 0.00002). To confirm our genotyping, we carried out Sanger sequencing (Eurofins) of a 2.4 kb region spanning (chr2:217,955,586–217,958,000) in two individuals who were heterozygous at the linked PRE2 SNP rs138522813. Primers were: forward 5′-CGCTTCCCCTTCATCACTTG-3′ and, reverse 5′-TCTCTCAGGCCAAGTCACAG-3′. Sequencing confirmed the presence of REF and ALT alleles of esv3594306, rs72951831, and rs199804270 (rs138522813 maps just outside the amplified region) but only REF alleles at rs572022984; on this basis we excluded rs572022984 from further analyses.

### Cloning of guides for CRISPR-based enhancer perturbation

Guides were designed using the online design tool CHOPCHOP (http://chopchop.cbu.uib.no). Guides were selected based on their proximity to variants of interest and specificity scores. Full details are provided in Table S3. Cloning was performed essentially as described in Ran et al.^19^ Briefly, guides were produced as two complementary oligonucleotides with overhangs to facilitate cloning. Oligos were annealed with T4 Polynucleotide Kinase (NEB). The expression vector pKLV-U6gRNA(BbsI)-PGKpuro2ABFP (Addgene #50946) was digested using BbsI (NEB), and ligation performed using T4 DNA ligase (NEB). Cloning was validated by sequencing (Eurofins Genomics).

### CRISPR-based enhancer perturbation

All CRISPR cell lines were derived from a parental MCF-7 cell line. Expression of each dCas9 construct was introduced by transduction with a specific Cas9-expressing lentivirus: pGH125_dCas9-Blast (Addgene #85417) for dCas9; pHR-SFFV-KRAB-dCas9-P2A-mCherry (Addgene #60954) for dCas9-KRAB; Lenti-hEF1-BLAST-dCas9-VPR (Dharmacon, CAS11916) for dCas9-VPR. Successfully transduced cells were then selected for by mCherry expression (dCas9-KRAB) or treatment with 10 μg/mL blasticidin (dCas9 and dCas9-VPR; GIBCO). Cells were then seeded into 24-well plates at a density of 50,000 cells per well. 100 μL of sgRNA lentivirus was added. After 24 h, media was replaced and after 48 h cells were lysed using the Cells-to-Ct kit (Life Technologies) for subsequent gene expression analysis by RT-PCR.

### Real-time PCR

Real-time PCR analysis of gene expression in cDNA samples was performed using Taqman probes (Life Technologies) for *IGFPB2* (MIM: 146731), *IGFBP5*, and *RPL37A* (MIM: 613314) normalized to the housekeeping gene *GAPDH* (ThermoFisher; *IGFBP2*: Hs01040719_m1, *IGFBP5*: Hs00181213_m1, *RPL37A*: Hs01102345_m1, *GAPDH*: Hs03929097_g1). Reactions of 5 μL were established using Taqman Universal Master Mix II, without UNG (Applied Biosystems) according to the manufacturer’s instructions.

### Statistical analysis of reporter gene assays and CRISPR-based enhancer perturbation

Firefly luciferase activity was internally normalized to renilla luciferase activity, and each test condition normalized to the “*IGFBP5* promoter-alone” (IGFBP5-PROM) construct. Setting IGFBP5-PROM to 1.0, for each putative enhancer-containing reporter gene construct we used t tests to test (1) H_0_: the mean dual luciferase ratio does not differ from 1.0 and (2) H_0_: the ALT construct does not differ from the REF construct. To compare mean dual luciferase ratios for each combination of SNP and SV at PRE2, we used three-way analysis of variance adjusting each variant for all other variants. To account for multiple testing, we used a Bonferroni corrected p value of 0.0056 (individual constructs, Figure 2; 9 tests) and 0.017 (PRE2 combinations, Figure 3; 3 tests).

Relative gene expression was calculated using the ΔΔC_T_ method. For the negative control sgRNAs (TAG-1 and TAG-2), we used t tests to test H_0_: the relative gene expression does not differ from 1.0. To maximize the power of subsequent analyses, we then combined the negative control data and for each of the other sgRNAs we tested H_0_: relative gene expression does not differ from the combined negative control relative gene expression. To account for multiple testing, we used a Bonferroni corrected p value of 0.017 (PROM sgRNAs Figures 4A; 3 tests per gene) and 0.0056 (PRE2 sgRNAs, Figures 4B and 4C; 9 tests per gene).

### Ethics approval and consent to participate

All participating studies were approved by their appropriate ethics review board and all subjects provided informed consent.

## Results

**Figure 1:**
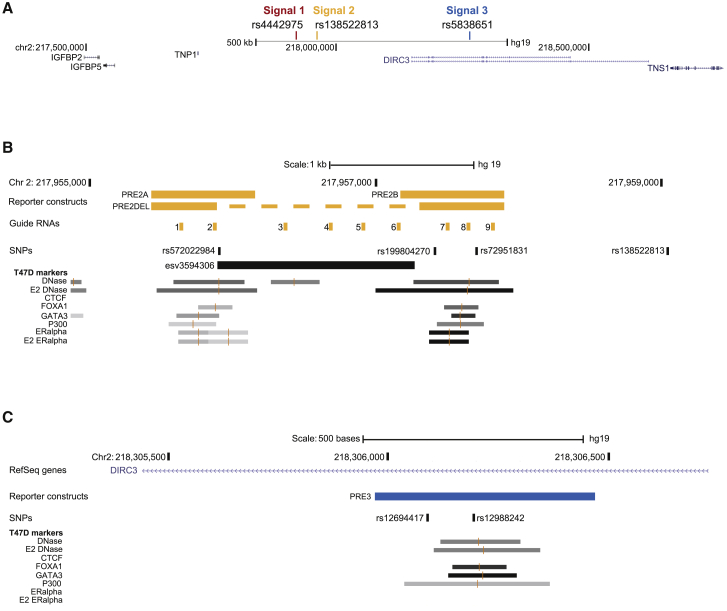
2q35 breast cancer risk locus (A) Fine-scale mapping at 2q35 identified three high-confidence (p < 1 × 10^−6^) signals annotated by rs4442975 (signal 1), rs138522813 (signal 2), and rs5838651 (signal 3). The putative target gene (*IGFBP5*) maps 360 kb, 399 kb, and 703 kb from signals 1, 2, and 3, respectively. All coordinates are based on GRCh37/hg19. (B) Putative regulatory element 2 (PRE2; chr2:217,955,458–217,957,767) at signal 2 colocalizes with four highly correlated variants: three single-nucleotide polymorphisms (SNPs; rs572022984, rs199804270, and rs72951831) and a 1.4 kb insertion/deletion variant (esv3594306; indicated by a black bar). A fourth SNP (rs138522813) maps outside the proposed boundaries of PRE2. Regions of open chromatin (DNase I) and ChIP-seq binding peaks for transcription factors are shown as gray bars where the shade of gray indicates the strength of the ChIP-seq peak (light gray, weak binding; dark gray, strong binding). Also shown (yellow bars) are the coordinates of three reporter gene constructs (PRE2A, PRE2B, and PRE2DEL) and the locations of sequences targeted by nine small guide (sg)RNAs. (C) PRE3 (chr2:218,305,944–218,306,443) indicated by a blue bar colocalizes with two SNPs (rs12694417 and rs12988242). Regions of open chromatin and ChIP-seq binding peaks are as in (B).

Fine-scale mapping of a 1.4 Mb region at 2q35 (chr2:217,407,297–218,770,424; GRCh37/hg19; Figure 1A) in combined data from up to 109,900 individuals with breast cancer and 88,937 control subjects of European Ancestry from the Breast Cancer Association Consortium confirmed the presence of three independent signals (p < 5 × 10^−8^; Figure S2) at this region.^3^ After conditioning on the top SNP at each of these three signals (signal 1, rs4442975; signal 2, rs138522813; signal 3, rs5838651), there were no additional high-confidence signals (defined as signals for which p < 1 × 10^−6^).^3^ Defining credible causal variants at each signal as variants with conditional p values within two orders of magnitude of the index variant there were 1, 5, and 42 credible causal variants at PRE1, PRE2, and PRE3, respectively (Table S4). Fine-scale mapping of this region in women of Asian Ancestry (12,481 affected individuals and 12,758 control subjects) did not identify any population-specific signals (all associations p > 5 × 10^−8^; Figure S3). None of the credible causal variants at signal 2 was present in women of Asian ancestry. The published causal variant at signal 1 (rs4442975) and all of the signal 3 credible causal variants (Table S5) were nominally associated with breast cancer risk in Asian women (p < 0.05). At signal 3, the index variants differ between Europeans and Asians (rs5838651 and 2:218265091:G:<INS:ME:ALU>:218265367, respectively) but none of the European credible causal variants could be excluded on the basis of the Asian data.

The T-allele of rs4442975 was associated with reduced breast cancer risk (per allele OR = 0.88, 95% CI 0.87–0.89, p = 1.3 × 10^−75^ and OR = 0.94, 95% CI 0.89–1.00, p = 0.04 in European and Asian women, respectively) and the delG-allele of rs5838651 was associated with increased risk (per allele OR = 1.07, 95% CI 1.05–1.08, p = 1.5 × 10^−16^ and OR = 1.07, 95% CI 1.03–1.11, p = 0.0008 in European and Asian women, respectively; Table 1). The delT-allele of rs138522813 was associated with reduced risk (carrier OR = 0.80 95% CI 0.77–0.83, p = 5.5 × 10^−32^). Stratifying by ER status, the signal 1 (rs4442975) and signal 2 (rs138522813) SNPs were more strongly associated with ER^+^ disease; for the signal 3 SNP (rs5838651), there was no evidence that the ORs differed by ER status (Table S6).

**Table 1:** Association of rs4442975, rs138522813 and rs5838651 among women of European and Asian ancestry

	**iCOGS**						**Oncoarray**					**Combined**							
	**MAF**^a^	**Cases**	**Controls**	**OR**^b^	**95% CI**	**P**_**1**_^c^	**MAF**	**Cases**	**Controls**	**OR**	**95% CI**	**P**_**1**_	**Cases**	**Controls**	**OR**	**95% CI**	**P**_**1**_	**P**_**het1**_^d^	**P**_**het2**_^e^
**Europeans**																			

rs4442975	0.49	36,471	37,251	0.88	0.86–0.89	4.9 × 10^−35^	0.48	57,920	46,226	0.88	0.87–0.90	1.7 × 10^−42^	94,391	83,477	0.88	0.87–0.89	1.3 × 10^−75^	0.46	0.49
rs138522813^f^	0.035	–	–	0.81	0.76–0.86	2.2 × 10^−12^	0.03	–	–	0.79	0.75–0.83	3.0 × 10^−21^	–	–	0.80	0.77–0.83	5.5 × 10^−32^	0.62	0.035
rs5838651	0.3	–	–	1.07	1.05–1.10	4.2 × 10^−9^	0.3	–	–	1.06	1.04–1.08	4.6 × 10^−9^	–	–	1.07	1.05–1.08	1.5 × 10^−16^	0.40	0.3

**Asians**																			

rs4442975	0.87	4,994	5,866	0.96	0.88–1.04	0.29	0.88	7,487	6,892	0.93	0.87–1.01	0.07	12,481	12,758	0.94	0.89–1.00	0.04	0.68	0.02
rs138522813^f^	–	–	–	–	–	–	–	–	–	–	–	–	–	–	–	–	–	–	–
rs5838651	0.61	–	–	1.03	0.97–1.10	0.29	0.62	–	–	1.09	1.04–1.14	0.0005	–	–	1.07	1.03–1.11	0.0008	0.18	0.95

a MAF, minor allele frequency

b OR, per allele odds ratio

c P_1_, test of H_0_ no association between SNP and breast cancer risk

d P_het1_, test of H_0_ no difference between iCOGS and OncoArray data

e P_het2_, test of H_0_ no difference between European and Asian data

f rs138522813-Del allele is extremely rare in Asians (MAF ~0.05%) and was not analyzed in Asian data

### Prioritization of credible variants for functional follow up

Fachal and colleagues^3^ used a Bayesian approach (PAINTOR) that combines genetic association, linkage disequilibrium, and enriched genomic features to determine variants with high posterior probabilities of being causal (Table S4).^20^ rs4442975, the only credible causal variant at signal 1 (posterior probability = 0.84), has previously been proposed to have a functional effect on breast cancer risk.^11^^,^^12^ Four of the five variants at signal 2 had posterior probabilities ≥ 0.20 (combined posterior probability 0.997); none of the variants at signal 3 had posterior probabilities > 0.15. To further prioritize putative causal variants at signals 2 and 3, we aligned the 47 credible variants at these signals with markers of open chromatin (DNase I), active transcription (P300), active enhancers (H3K27Ac, H3K4me1), and breast-relevant TFs (FOXA1, GATA3, ERα) generated in T-47D and MCF-7 breast cancer cells^15^, ^16^, ^17^ (Table S4). Consistent with the PAINTOR posterior probabilities, four variants at signal 2 colocalized with at least one of these features. In addition, we identified two variants at signal 3 that colocalized with one of these features. These six variants were prioritized for further functional annotation.

### Reporter gene assays of prioritized variants

**Figure 2:**
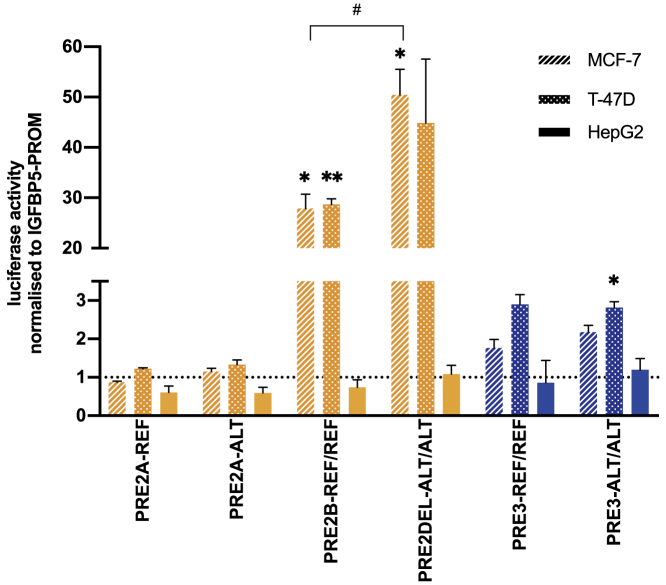
Luciferase reporter assays following transient transfection of PRE2 and PRE3, REF and ALT constructs, into MCF-7, T-47D, and HepG2 cells The PRE containing the reference (REF) allele at each SNP was cloned downstream of the *IGFBP5* promoter to generate reference (REF) luciferase constructs. Alternative (ALT) alleles were generated by site-directed mutagenesis. Coordinates of the PREs are given in Table S2, diagrams are in Figure S1. Error bars denote standard deviations based on three independent experiments each done in triplicate. p values were determined by t tests and a Bonferroni correction was applied to account for multiple testing. Comparing each PRE containing construct to *IGFBP5*-PROM, ^∗^p < 0.0056, ^∗∗^p ≤ 0.00056; comparing ALT to REF constructs ^#^p < 0.0056.

For SNPs, we generated reference (REF) and alternative (ALT) constructs in which the putative regulatory element, defined in the first instance as a 500 to 700 bp region centered on the SNP or SNP pair (PRE2A rs572022984; PRE2B rs199804270 and rs72951831; PRE3 rs12694417 and rs12988242, Table S2; Figures 1B and 1C), was cloned upstream of a luciferase reporter gene, driven by the *IGFBP5* promoter (Figure S1). For the structural variant esv3594306, which is defined by the presence (REF) or absence (ALT) of a 1.4 kb region (chr2:217,955,891–217,957,273; GRCh37/hg19), we generated separate REF constructs for PRE2A and PRE2B and a single ALT construct in which the centromeric sequences at PRE2A were juxtaposed to the telomeric sequences at PRE2B with the intervening 1.4 kb deleted (Figure 1B). Comparing the REF construct at each region with the *IGFBP5* promoter construct (IGFBP5-PROM), there was evidence that two of the putative regulatory elements (PRE2B and PRE3) enhanced transcription from the *IGFBP5* promoter (Figure 2). For PRE2B, both alleles demonstrated strong enhancer activity (PRE2B-REF/REF: fold change [FC] = 27.9, p = 0.004 and FC = 28.7, p = 0.0005; PRE2DEL-ALT/ALT: FC = 50.5, p = 0.004 and FC = 44.9, p = 0.03 in MCF-7 and T-47D, respectively). For PRE3 the activity was more modest and only significant (p < 0.0056; Material and methods) for the ALT allele in T-47D (PRE3-REF/REF: FC = 1.8, p = 0.03 and FC = 2.9, p = 0.006; PRE3-ALT/ALT FC = 2.2, p = 0.008 and FC = 2.8, p = 0.003 in MCF-7 and T-47D, respectively; Figure 2). To test these constructs for cell type specificity, we used HepG2 (hepatocyte carcinoma), 293T (embryonic kidney), and HCT116 (colorectal carcinoma) cells; the only construct that influenced transcription from the *IGFBP5* promoter in these non-breast cells was PRE2DEL-ALT/ALT in 293T cells and with an effect size that was an order of magnitude lower (FC = 1.9, p = 0.002; Figure S4) compared to the breast cancer cell lines (FC > 40; Figure 2). Comparing ALT constructs with REF constructs, only the PRE2 region showed a significant difference between alleles, with the (protective) PRE2DEL-ALT/ALT allele being associated with greater activity than PRE2B-REF/REF allele (MCF-7 FC = 1.8, p = 0.003; T-47D FC = 1.6, p = 0.09; Figure 2). Repeating these assays in cells that were grown in the presence of low-dose estradiol did not alter these results; both PRE2B and PRE3 were responsive to low-dose estradiol (Figures S5A and S5B) but only PRE2 showed a difference between alleles, with the protective PRE2DEL-ALT/ALT allele once again being associated with significantly greater activity than the PRE2B-REF/REF allele, this time in T-47D cells (MCF-7 FC = 1.5, p = 0.15; T-47D FC = 2.7, p = 0.002; Figure S5A).

**Figure 3:**
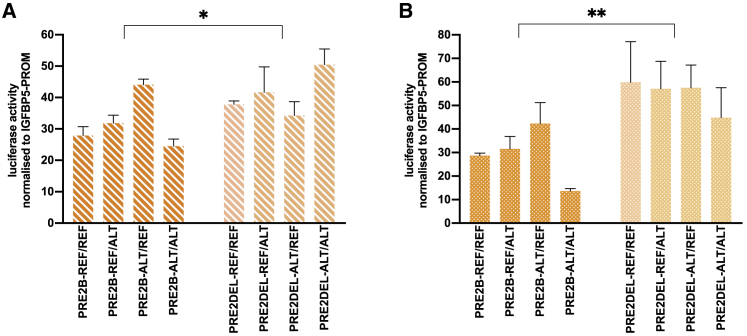
Luciferase reporter assays following transient transfection of constructs with allelic variants at PRE2B and PRE2DEL into MCF-7 and T-47D cells Reporter gene constructs with all possible combinations of rs199804270 and rs72951831 and esv3594306 were generated by site-directed mutagenesis of the naturally occurring haplotypes at PRE2B and PRE2DEL (Material and methods) into MCF-7 (A) and T-47D (B) cells. Coordinates of the PREs are given in Table S2, diagrams are in Figure S1. Error bars denote standard deviations based on three independent experiments each done in triplicate. 3-way ANOVA was used to compare each variant, adjusted for the other two variants, a Bonferroni correction was applied to account for multiple testing. ^∗^p < 0.017, ^∗∗^p ≤ 0.0017.

The PRE2DEL-ALT/ALT construct comprises a haplotype of three tightly linked variants: the ALT alleles of the two SNPs (rs199804270:GA:G, rs72951831:G:T) with the ALT (deletion) allele of the structural variant (esv3594306) that brings two separate ERα, FOXA1, GATA3, and P300 ChIP-seq peaks into juxtaposition (Figure 1B). To differentiate individual effects, each allele of each SNP was introduced onto esv3594306 insertion and deletion backgrounds separately using site-directed mutagenesis. The PRE2A SNP (rs572022984) was not considered further due to technical issues (Material and methods). In a combined analysis, adjusting each variant for the other two variants, there was evidence that deletion constructs consistently showed greater activity than insertion constructs (MCF-7: DEL FC = 43.4, INS FC = 34.4, i.e., average additional FC for DEL = 1.3, p_het_ = 0.01; T-47D: DEL FC = 47.3, INS FC = 21.6, i.e., average additional FC for DEL = 2.2, p_het_ = 1.7 × 10^−8^; Figure 3).

### CRISPR-based perturbation of PRE2

Reporter gene assays do not reflect the “normal” genomic context of a regulatory element. Specifically, the assay tests whether the putative regulatory element can influence expression in an episomal context^21^ and from a distance of a few kilobases; *in vivo*, PRE2 maps approximately 400 kb from the *IGFBP5* promoter. To determine whether PRE2 acts as an enhancer element in a cellular context, we used a systematic CRISPR-based enhancer perturbation approach. We hypothesized that if PRE2 acts as an enhancer *in vivo*, targeting a catalytically inactive Cas9 (dCas9) fused to a repressive (KRAB) domain to regions within PRE2 would result in lower levels of expression of *IGFBP5* (CRISPR interference; CRISPRi); by contrast, targeting dCas9 fused to an activating VPR domain would result in higher levels of expression of *IGFBP5* (CRISPR activation; CRISPRa).^22^^,^^23^ We designed CRISPR single-guide (sg)RNAs to the ERα ChIP-seq peak at the centromeric breakpoint of the deletion (guides PRE2-1 and -2), within the esv3594306 deletion region (guides PRE2-3 to -6) and to the ERα ChIP-seq peak at the telomeric breakpoint of the deletion (guides PRE2-7 to -9; Figure 1B). As positive controls we designed sgRNAs to target the *IGFBP5* promoter (guides PROM-1 to -3; Figure S6A) and the previously characterized causal variant (rs4442975, guide PRE1-1; Figure S6B). As negative controls we designed sgRNAs to the published genome-wide association study signal 1 tag SNP (rs13387042, guides TAG-1 and -2; Figure S6B). We used MCF-7 cell lines engineered to stably express (1) dCas9 with a repressive KRAB domain and (2) dCas9 with an activating VPR domain; as an additional control we used MCF-7 cells that expressed dCas9 without the KRAB or VPR domains.

**Figure 4:**
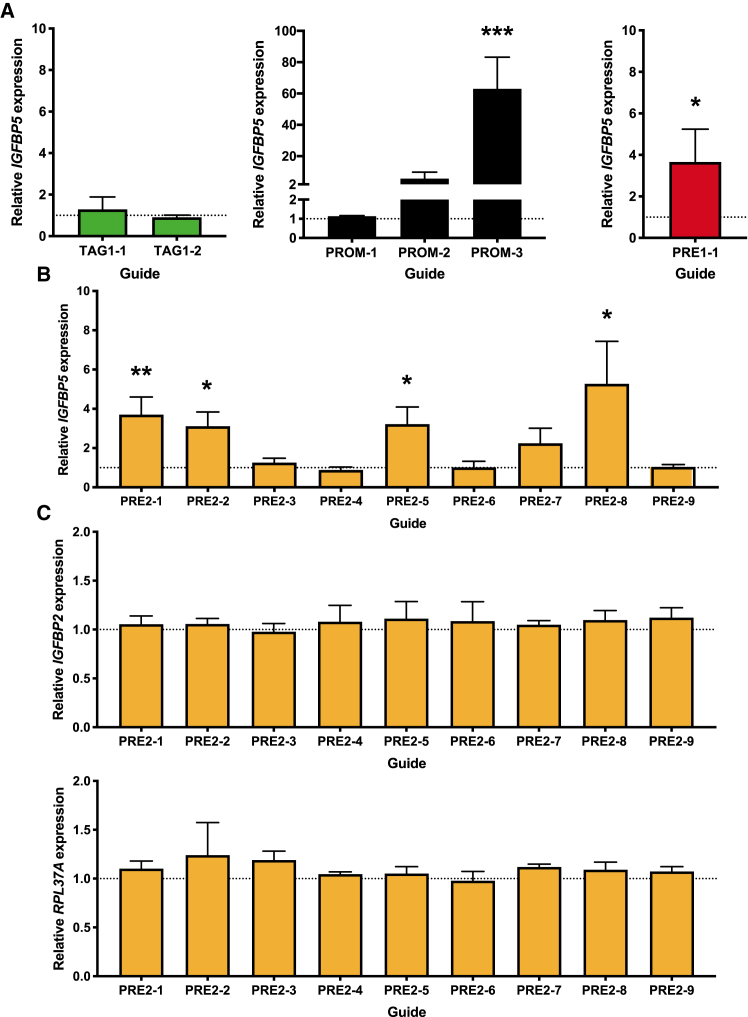
Systematic CRISPRa analysis of 2q35 putative regulatory elements MCF-7 cells expressing dCas9-VPR were transduced with CRISPR sgRNAs targeting: (A) the PRE1 tag SNP rs13387042 (negative control), the *IGFBP5* promoter and the PRE1 causal variant rs4442975 (positive control), and (B and C) a series of sites mapping across PRE2 (Figure 1B). Relative gene expression (compared to vector alone) was calculated using the ΔΔC_T_ method. Full details of guide RNAs are listed in Table S3. Error bars denote standard deviations based on three independent experiments each done in triplicate. p values were determined by t tests and a Bonferroni correction was applied to account for multiple testing; (A) ^∗^p < 0.017, ^∗∗^p < 0.0017, ^∗∗∗^p < 0.00017; (B and C) ^∗^p < 0.0056, ^∗∗^p ≤ 0.00056.

**Figure 5:**
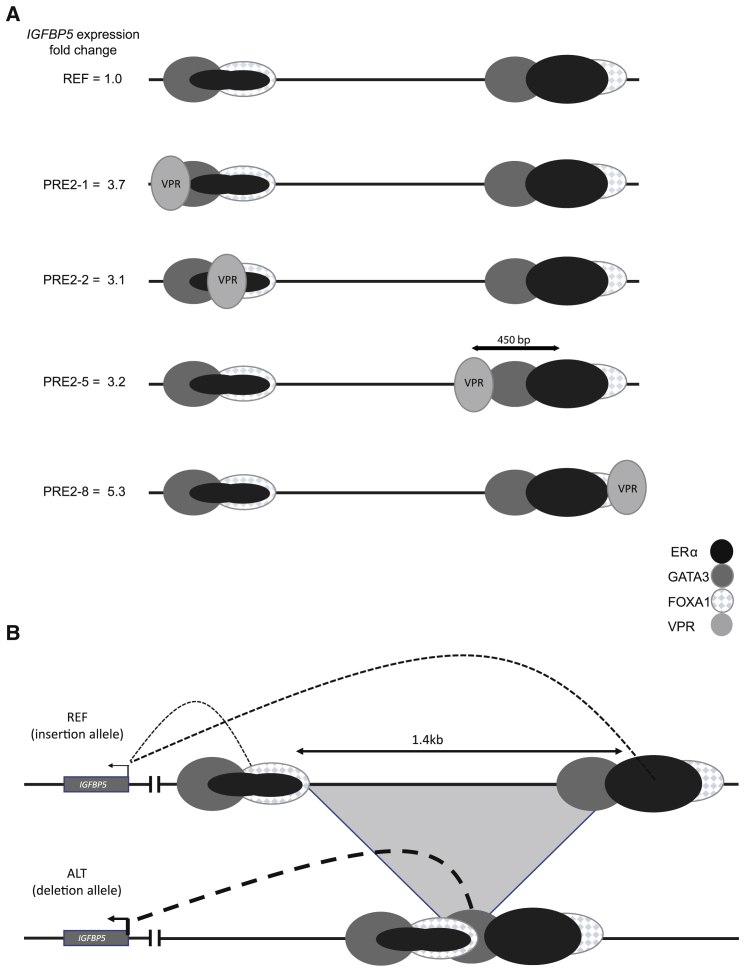
Increasing the local density of activator TF domains with dCas9-VPR or by juxtaposition of two ChIP-seq peaks is associated with increased expression of *IGFBP5* (A) Introducing dCas9 fused to a VPR activator domain at the ERα, FOXA1, GATA3 ChIP-seq peak at the centromeric end of the deletion breakpoint (PRE2-1 and PRE2-2), proximal to, or at, the ERα, FOXA1, GATA3 ChIP-seq peak at the telomeric end of the deletion breakpoint (PRE2-5 and PRE2-8, respectively) increases expression of *IGFBP5* in MCF-7 cells. (B) Deletion of 1.4 kb on the ALT allele of esv3594306 juxtaposes these two ERα, FOXA1, GATA3 ChIP-seq peaks. In each case (A and B) this increases the density of activating TF domains in the region and is associated with increased expression of *IGFBP5*.

In the dCas9 cell line, there was just one sgRNA (PROM-2) that influenced *IGFBP5* expression; this sgRNA targets the *IGFBP5* promoter, colocalizing with the transcription start site (TSS) and likely reduces expression of *IGFBP5* by steric hindrance (60% reduction, p = 0.004; Figure S7A). In the CRISPRi setting, all three sgRNAs targeting the *IGFBP5* promoter repressed *IGFBP5* expression significantly to 8%–15% of levels in the negative controls (p = 0.001, p = 0.001, and p = 0.0008 for guides PROM-1, -2, and -3, respectively; Figure S8A). No sgRNA targeting non-promoter sequences influenced *IGFBP5* expression (Figures S8A and S8B). In the CRISPRa setting, the sgRNA 5′ to the *IGFBP5* promoter (PROM-3; Figure 4A) enhanced *IGFBP5* expression more than 60-fold (p = 0.00008) and the PRE-1-positive control sgRNA (PRE1-1) targeting rs442975 also enhanced *IGFBP5* expression (FC = 3.7, p = 0.006; Figure 4A). In addition, four of the nine sgRNAs targeting sequences at PRE2 enhanced *IGFBP5* expression; specifically PRE2-1 and -2 targeting the ERα ChIP-seq peak at the centromeric deletion breakpoint (PRE2-1: FC = 3.7, p = 0.0005; PRE2-2: FC = 3.1, p = 0.001), PRE2-5 at the distal end of the deletion region (PRE2-5: FC = 3.2, p = 0.002), and PRE2-8 targeting the ERα ChIP-seq peak immediately telomeric to the deletion region (PRE2-8: FC = 5.3, p = 0.002; Figures 4B and 5A). None of the sgRNAs influenced expression of two genes mapping immediately 3′ to *IGFBP5* (*IGFBP2* and *RPL37A*; Figure 4C).

## Discussion

Fine-scale mapping at the 2q35 breast cancer locus in women of European ancestry^3^ confirmed rs4442975 as the probable causal variant at signal 1 and reduced the number of credible causal variants at signal 2 from 14 to 5;^3^^,^^14^ at signal 3, however, there remained 42 credible causal variants that could not be excluded as causal on statistical grounds alone in either the European or the Asian data. Low-throughput functional approaches that are used to investigate putative causal variants, including reporter gene assays and CRISPR screens, become prohibitive with large numbers of credible causal variants and most single locus^11^^,^^14^^,^^24^, ^25^, ^26^, ^27^, ^28^, ^29^, ^30^, ^31^, ^32^, ^33^, ^34^, ^35^, ^36^, ^37^, ^38^ and global^3^^,^^6^ annotation studies have used co-localization of credible causal variants with markers of open chromatin, active histone modifications, and transcription factor binding in relevant cell types to prioritize credible causal variants for functional follow up. Of the 811 annotation tracks that were examined in a recent global fine-scale mapping analysis,^3^ credible causal variants were enriched at three types of genomic features that are relevant to long-range regulatory elements: (1) open chromatin in ER^+^ cell lines and normal breast, (2) the active histone marks H3K4me1 and H3K27ac in MCF-7 cells, and (3) ESR1, FOXA1, GATA3, and P300 TF binding sites. By aligning the five credible causal variants at PRE2 and the 42 credible causal variants at PRE3 with these marks (Table S4), we were able to prioritize 4 of the 5 credible causal variants at PRE2 and 2 of the 42 credible causal variants at PRE3 for follow-up studies. By taking this approach there is, inevitably, the possibility that we have excluded one or more causal variants from our follow-up analyses. For PRE2 this seems unlikely as we selected four out of the five credible causal variants for further follow-up studies. For PRE3 it is entirely possible, or even probable, that we failed to prioritize one or more causal variant(s); improving our ability to discriminate more accurately between potentially functional variants and large numbers of correlated variants will require genome-wide datasets with functional outputs^21^^,^^39^^,^^40^ generated in more relevant cellular disease models and taking advantage of single-cell technologies.^1^

Using reporter gene assays, we have demonstrated that both the distal region of PRE2 (PRE2B) and the entire PRE3 region can enhance transcription from the *IGFBP5* promoter in a cell-type-specific manner. Despite co-localizing with multiple markers, we found no evidence that the proximal region of PRE2 (PRE2A) acts as an independent enhancer element. The ChIP-seq peaks at this region are, however, relatively weak (Figure 1B); combining data from both PRE2A alleles, in both breast cancer cell lines to increase our power (i.e., using 12 replicates rather than 3) the overall mean fold change for PRE2A was 1.14 (1.03–1.26, p = 0.01), consistent with the presence of a very modest enhancer element. Comparing REF constructs with ALT constructs, we found no evidence that either of the credible causal variants at PRE3 (rs12694417, rs12988242) altered the activity of the PRE. This does not exclude these SNPs as functional; as above, modest effects on enhancer activity may be difficult to detect and variants that, for example, influence chromatin accessibility may not be detectable in transient assays.^11^ However, without preliminary *in vitro* evidence to suggest that one of these variants alters cell-type-specific transcription from the *IGFBP5* promoter, pursuing further functional studies that are predicated on this very assumption seems unlikely to be fruitful. By contrast, one comparison that was consistent and significant between constructs and across the two breast cancer cell lines was that PRE2 deletion alleles had stronger enhancer activity than PRE2 insertion alleles.

The purpose of our CRISPR-based enhancer perturbation was 2-fold: specifically, to interrogate the PRE2 region within its normal genomic context and more generally to evaluate CRISPRi and CRISPRa approaches for interrogating long-range regulatory elements that harbor credible causal variants. As none of our PRE2 sgRNAs impacted *IGFBP5* expression significantly in the CRISPRi setting, our analysis raises questions as to the utility of this approach for characterizing long-range regulatory elements (PRE2 maps approximately 400 kb telomeric to the *IGFBP5* promoter). This is at odds with results of a systematic CRISPRi screen to identify enhancer elements in K562 cells, which demonstrated CRISPRi-mediated repression of c-MYC expression by sgRNAs targeting sequences mapping up to 1.9 Mb downstream of c-MYC.^22^ In this analysis, however, CRISPRi-mediated repression by these distal elements was modest compared to CRISPRi-mediated repression by more proximal elements and, even based on 12 biological replicates, of borderline statistical significance.^22^ By contrast, using CRISPRa we were able to confirm that one or more elements within PRE2 can act as a long-range regulatory element that specifically targets *IGFBP5* (rather than *IGFBP2* or *RPL37A*). Four of the nine guide RNAs targeting dCas9-VPR to sequences at PRE2 increased expression of *IGFBP5*; three of these colocalized with ERα, FOXA1, and GATA3 ChIP-seq peaks (PRE2-1, -2, and -8) and a fourth (PRE2-5) mapped within the esv3594306 deleted region (Figure 5A). There were also two guides which targeted dCas9-VPR to sequences that map close to the distal ERα, FOXA1, and GATA3 ChIP-seq peak (PRE2-6 and -7) but did not increase *IGFBP5* expression; this may reflect the very variable efficiency of different guide RNAs.^22^ We present a theoretical model in which we hypothesize that all of the PRE2 guides that increased expression of *IGFBP5* increased the local density of activating TF domains by bringing a VPR domain into the proximity of a cluster of TF ChIP-seq peaks; one implication of the increase in *IGFBP5* expression we observed with PRE2-5, which maps approximately 450 bp from the center of the nearest cluster of ChIP-seq peaks (Figure 5A), is that these regulatory elements may extend over relatively large (>1 kb) regions. This should not, perhaps, be surprising; at a subset of strongly activated E2-responsive enhancers, it has previously been shown that ERα recruits DNA-binding transcription factors in *trans*, to form a large (1–2 MDa) complex.^41^

It has previously been suggested that sequences mapping to PRE2 act as a repressor element which, in the presence of low-dose estradiol, acts to reduce *IGFBP5* expression.^14^ By contrast, our data support PRE2 acting as a powerful enhancer element with the deletion allele increasing expression of *IGFBP5* over and above that of the insertion allele with or without estradiol stimulation. Overall, our data are consistent with a hypothetical model in which the juxtaposition of the two ERα, FOXA1, GATA3 binding sites at PRE2 by deletion of approximately 1.4 kb of intervening sequence generates a single extended binding region (Figure 5B) that is causally associated with increased enhancer activity, higher levels of expression of the putative tumor suppressor gene *IGFBP5*,^42^ and a reduction in breast cancer risk (OR = 0.77, p = 2.2 × 10^−29^) that is largely restricted to ER^+^ disease.

In conclusion, we have identified putative enhancer elements at two additional 2q35 breast cancer risk loci. One of these, mapping approximately 400 kb telomeric to *IGFBP5*, enhances transcription from the *IGFBP5* promoter by a factor of 30- to 40-fold. For this element we provide evidence that a deletion of 1.4 kb is causally associated with increased enhancer activity and suggest a mechanism for this increased activity.

## Consortia

The NBCS Collaborators are Anne-Lise Børresen-Dale, Grethe I. Grenaker Alnæs, Kristine K. Sahlberg, Lars Ottestad, Rolf Kåresen, Ellen Schlichting, Marit Muri Holmen, Toril Sauer, Vilde Haakensen, Olav Engebråten, Bjørn Naume, Alexander Fosså, Cecile E. Kiserud, Kristin V. Reinertsen, Åslaug Helland, Margit Riis, Jürgen Geisler, and OSBREAC.

The kConFab Investigators are David Amor, Lesley Andrews, Yoland Antill, Rosemary Balleine, Jonathan Beesley, Ian Bennett, Michael Bogwitz, Leon Botes, Meagan Brennan, Melissa Brown, Michael Buckley, Jo Burke, Phyllis Butow, Liz Caldon, Ian Campbell, Deepa Chauhan, Manisha Chauhan, Georgia Chenevix-Trench, Alice Christian, Paul Cohen, Alison Colley, Ashley Crook, James Cui, Margaret Cummings, Sarah Jane Dawson, Anna deFazio, Martin Delatycki, Rebecca Dickson, Joanne Dixon, Ted Edkins, Stacey Edwards, Gelareh Farshid, Andrew Fellows, Georgina Fenton, Michael Field, James Flanagan, Peter Fong, Laura Forrest, Stephen Fox, Juliet French, Michael Friedlander, Clara Gaff, Mike Gattas, Peter George, Sian Greening, Marion Harris, Stewart Hart, Nick Hayward, John Hopper, Cass Hoskins, Clare Hunt, Paul James, Mark Jenkins, Alexa Kidd, Judy Kirk, Jessica Koehler, James Kollias, Sunil Lakhani, Mitchell Lawrence, Geoff Lindeman, Lara Lipton, Liz Lobb, Graham Mann, Deborah Marsh, Sue Anne McLachlan, Bettina Meiser, Roger Milne, Sophie Nightingale, Shona O'Connell, Sarah O'Sullivan, David Gallego Ortega, Nick Pachter, Briony Patterson, Amy Pearn, Kelly Phillips, Ellen Pieper, Edwina Rickard, Bridget Robinson, Mona Saleh, Elizabeth Salisbury, Christobel Saunders, Jodi Saunus, Rodney Scott, Clare Scott, Adrienne Sexton, Andrew Shelling, Peter Simpson, Melissa Southey, Amanda Spurdle, Jessica Taylor, Renea Taylor, Heather Thorne, Alison Trainer, Kathy Tucker, Jane Visvader, Logan Walker, Rachael Williams, Ingrid Winship, and Mary Ann Young.

The ABCTB Investigators are Christine Clarke, Deborah Marsh, Rodney Scott, Robert Baxter, Desmond Yip, Jane Carpenter, Alison Davis, Nirmala Pathmanathan, Peter Simpson, Dinny Graham, and Mythily Sachchithananthan.

## Declaration of interests

M.W.B. conducts research funded by Amgen, Novartis, and Pfizer. P.A.F. conducts research funded by Amgen, Novartis, and Pfizer and received honoraria from Roche, Novartis, and Pfizer. A.W.K. received research funding to her institution from Myriad Genetics for an unrelated project (funding dates 2017-2019). U.M. has stockownership in Abcodia Ltd. All other authors declare no conflict of interest.

## Data and code availability

Summary results for all variants genotyped by the Breast Cancer Association Consortium BCAC (including rs45446698) are available at http://bcac.ccge.medschl.cam.ac.uk/. Requests for data can be made to the corresponding author or the Data Access Coordination Committee (DACC) of the Breast Cancer Association Consortium via email to BCAC@medschl.cam.ac.uk.

## Web resources

1000 Genomes Project, https://www.internationalgenome.org/

## Supplemental information

Document S1. Figures S1–S8, acknowledgments, and funding details

Data S1. Tables S1–S6

Document S2. Article plus supplemental information
